# Evidence of a positive association between malpractice climate and thyroid cancer incidence in the United States

**DOI:** 10.1371/journal.pone.0199862

**Published:** 2018-07-18

**Authors:** Brandon Labarge, Vonn Walter, Eugene J. Lengerich, Henry Crist, Dipti Karamchandani, Nicole Williams, David Goldenberg, Darrin V. Bann, Joshua I. Warrick

**Affiliations:** 1 College of Medicine, The Pennsylvania State University, Hershey, PA, United States of America; 2 Department of Public Health Sciences, College of Medicine, The Pennsylvania State University, Hershey, PA, United States of America; 3 Department of Biochemistry, College of Medicine, The Pennsylvania State University, Hershey, PA, United States of America; 4 Penn State Cancer Institute, Hershey, PA, United States of America; 5 Department of Pathology, College of Medicine, The Pennsylvania State University, Hershey, PA, United States of America; 6 Department of Surgery–Division of Otolaryngology, College of Medicine, The Pennsylvania State University, Hershey, PA, United States of America; Stony Brook University, Graduate Program in Public Health, UNITED STATES

## Abstract

The incidence of thyroid cancer has risen dramatically in the past few decades. The cause of this is unclear, but several lines of evidence indicate it is largely due to overdiagnosis, the diagnosis of tumors that would have never manifest clinically if untreated. Practices leading to overdiagnosis may relate to defensive medicine. In this study, we evaluated the association between malpractice climate and incidence of thyroid, breast, prostate, colon, and lung cancer in U.S. states from 1999–2012 using publicly available government data. State-level malpractice risk was quantified as malpractice payout rate, the number of malpractice payouts per 100,000 people per state per year. Associations between state-level cancer incidence, malpractice payout rate, and several cancer risk factors were evaluated. Risk factors included several social determinants of health, including factors predicting healthcare access. States with higher malpractice payout rate had higher thyroid cancer incidence, on both univariate analysis (r = 0.51, P = 0.009, Spearman) and multivariate analysis (P<0.001, multilevel model). In contrast, state-level malpractice payout rate was not associated with incidence of any other cancer type. Malpractice climate may be a social determinant for being diagnosed with thyroid cancer. This may be a product of greater defensive medicine in states with higher malpractice risk, which leads to increased diagnostic testing of patients with thyroid nodules and potential overdiagnosis. Alternatively, malpractice risk may be a proxy for another, unmeasured risk factor.

## Introduction

The incidence of thyroid cancer has increased dramatically in the United States in the past several decades, from 3.6 cases per 100,000 in 1973 to 15 cases per 100,000 in 2014[1]. It is unclear what has caused this. Environmental exposures have been studied, but no specific exposure has been consistently linked to thyroid cancer incidence to date[2, 3]. Conversely, several lines of evidence suggest overdiagnosis, the diagnosis of tumors that would not have manifest clinically if left undetected, is largely responsible. This evidence includes a miniscule change in thyroid cancer mortality[1], a high incidence of thyroid cancer at *post mortem* examination[4], and increasing use of diagnostic ultrasound and fine needle aspiration (FNA) by physicians in patients with thyroid nodules[5], which detects these clinically indolent tumors. If overdiagnosis is primarily responsible for the increasing incidence, however, it is unclear what has driven physicians to increasingly test thyroid nodules. In contrast to breast and prostate cancer in the United States, and thyroid cancer in South Korea[6], where cancer screening programs have driven increased testing and overdiagnosis, screening for thyroid cancer is not practiced in the United States[7].

The strongest risk factors for thyroid cancer are social and geospatial. That is, thyroid cancer is more common in people with higher socioeconomic status[8, 9], and in those residing in northeastern states near the Atlantic Ocean, particularly Pennsylvania, New Jersey, and New York State[10]. People with higher socioeconomic status tend to have greater access to healthcare services, increasing contact with the healthcare system and risk of overdiagnosis, likely explaining the positive association between socioeconomic status and thyroid cancer risk. However, while the association with socioeconomic status has a reasonable explanation, the geospatial association with thyroid cancer incidence is largely unexplained.

A growing body of evidence indicates cancer risk is strongly associated with several social determinants of health. These broadly include socioeconomic status, physical environment, education, access to food, social context, and access and utilization of the healthcare system[11]. One potential social determinant of health that is largely unexplored is legal climate, specifically the legal climate created by malpractice tort. Tort law substantially influences the practice of medicine. Its effects are most visible as differences among states, in large part because tort law is regulated by state governments. Pennsylvania, New Jersey, and New York State are among the states with the highest number of tort rulings against physicians[12]. Tort law influences medical care partly through motivating the practice of “defensive medicine,” the practice of recommending tests or treatments that serve not to benefit the patient, but to protect the physician from being sued. The effects of defensive medicine are diverse. For example, obstetricians who practice in areas with higher tort risk perform more cesarean sections[13]. Neurosurgeons who practice in states with higher tort risk are more likely to practice “assurance” behaviors, such as ordering defensive laboratory tests[14]. In the practice of pathology, survey-based studies have shown fear of litigation drives many pathologists to favor malignant diagnoses in borderline lesions[15, 16]. The evidence indicates defensive practices are employed by physicians from diverse specialties, and have a meaningful impact on patient care.

In this paper, we investigate malpractice climate in The United States as a potential social determinant of thyroid cancer risk. We show that states with higher rate of malpractice payout have higher incidence of thyroid cancer, when controlling for healthcare access and other cancer risk factors. The findings suggest malpractice climate may be a social determinant of being diagnosed with thyroid cancer.

## Materials and methods

### Data collection

We collected data from all 50 states and the District of Columbia (DC) for the years 1999–2012. Malpractice payout data were obtained from the National Practitioner’s Data Bank (NPDB) (19). The NPDB reports the number of malpractice payouts per state per year, with payouts defined as the number of payments resulting from either settlement or judgement. The “malpractice payout rate” was determined as malpractice payout number per 100,000 people in each state in each year, with populations taken from The United States Census Bureau database[17].

Unadjusted (for age) thyroid, breast, prostate, colon, and lung cancer incidence data were obtained from the Centers for Disease Control and Prevention (CDC) United States Cancer Statistics (USCS) database[18]. Behavioral and race data were collected from the CDC Behavioral Risk Factor Surveillance System (BRFSS)[19]. These included percentage of population under 65 years of age with health insurance (“insurance”), percentage of the population identifying as Caucasian (“race”), percentage of individuals over the age of 18 years who are current smokers (“smoking”), and percentage of the population with a body mass index (BMI) >30 (considered obese; “obesity”). Median household income (“income”) and percentage of population in poverty (“poverty”) were obtained from the United States Census Bureau Income & Poverty Data Tables[20]. State-level age data were obtained from the United States Census Bureau database[17], and the variable “age” was defined as mean age of the population in each state in each year.

### Data analysis

#### Univariate analysis

**Fig 1:**
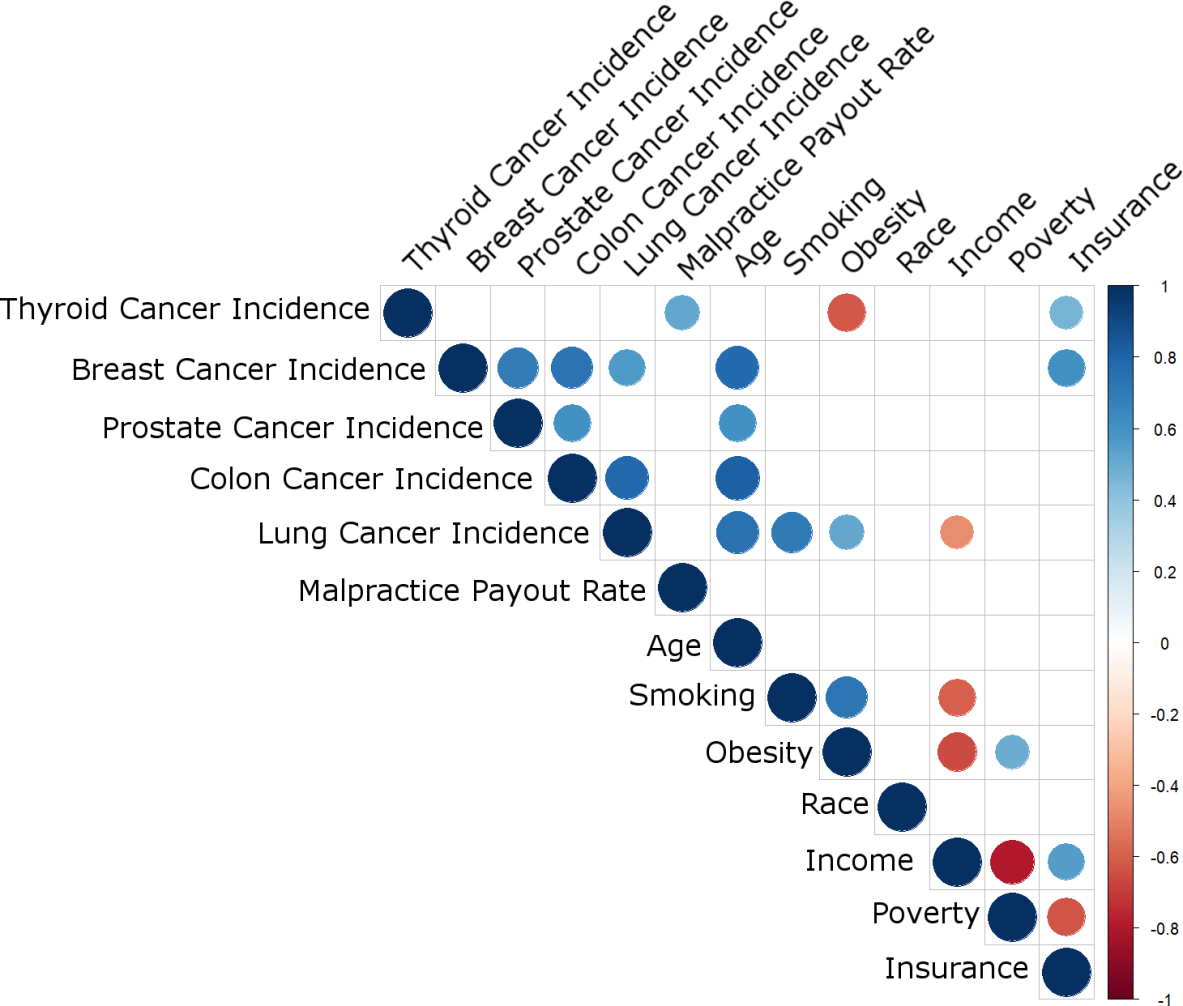
Correlation matrix including cancer incidences, malpractice payout rate, and cancer risk factors. Correlations are represented as colored circles, blue indicating positive correlation, and red indicating negative correlation. Larger circle size and greater color intensity indicate higher correlation coefficient (see color key). Blank space indicates the correlation was not statistically significant (Spearman correlation with Bonferroni correction, *P* < 0.05 considered significant). The complete correlation matrix is presented in **S1 Table**.

Univariate analysis was based on the average value of each variable in each state from all years under study. All variables, including cancer incidence and epidemiologic measures, were compared with a correlation matrix, using the Spearman method. All p-values were corrected with the Bonferroni method, to reduce the risk of Type 1 errors, and p<0.05 was considered significant. Figures were generated using R version 3.1.1[21]. Fig 1 was produced using the corrplot package in R[22].

#### Multivariate model design

We created five multivariate models, one for each cancer type. The models were constructed to evaluate the association between malpractice payout rate and incidence of each cancer type, among states, when controlling for other cancer risk factors. To create uniform models, which allowed direct comparison of different cancer types to one another, we designed the five models to include the same set of covariates.

In additional to issues of colinearity (see below), exploratory analyses identified two factors that we controlled for in our modeling approach. First, there were strong associations between state-level cancer incidence and calendar year. Second, mean cancer incidence from 1999–2012 varied considerably by state. These observations, combined with the fact that we had repeated measures of cancer incidence for each state (i.e. one measurement per cancer type for each year from 1999–2012), informed our decision to use a mixed effects model that included state-specific random intercepts. All remaining covariates were fixed effects.

High collinearity was observed among insurance, income, and poverty. We thus created a composite measure of these three variables, defined as “healthcare access,” and quantified as the mean of standardized versions of insurance, income, and 1-poverty. Smoking and obesity were highly colinear, prompting us to include only one of them in the final models (smoking). Race did not correlate with incidence of any cancer type on univariate analysis, or any other risk factor, and was thus excluded from the final models. Other than year, all covariates were time-dependent. Previous studies have shown that care must be taken when using time-dependent covariates in linear models, and using the method of Neuhaus and Kalbfleisch[23] we created “within” and “between” versions of these variables. Malpractice payout rate was log transformed, owing to a right skew. Malpractice payout rate, age, smoking, and year were centered. The final models included the covariates of year, malpractice payout rate, age, smoking, and healthcare access.

Initially we considered a mixed effects logistic regression model with a random intercept, but an analysis of the residuals suggested that a linear mixed model with a variance-stabilizing transformation yielded a better fit. We achieved this by applying $arcsin(\sqrt{x})$ to each cancer incidence to obtain our response variables. For both approaches we used the GLIMMIX procedure in SAS University Edition Software (SAS Institute, Cary, NC). All p-values were corrected with the Bonferroni method, to reduce the risk of Type 1 errors, and p<0.05 was considered significant.

## Results

### Variability of cancer incidence and cancer risk factors among states

The incidence of different cancer types varied among states. For example, thyroid cancer incidence was highest in Pennsylvania at 16.5 per 100,000 people, and lowest in Alabama at 7.6 cases per 100,000 people. Further details are presented in **Table 1**.

**Table 1:** Variation in cancer incidence among states.

	Mean Incidence	Standard Deviation	Range	Top Decile States	Bottom Decile States
Thyroid	11	2	7.6–16.5	PA, MA, RI, CT, NJ	AL, AR, OK, SC, GA
Breast	140	14	93–163	CT, ME, VT, PA, MA	UT, TX, AK, NV, NM
Prostate	151	19	99–185	MT, DE, DC, ME, ND	AK, TX, AZ, UT, IN
Colon	51	8	28–68	WV, PA, IA, ME, ND	UT, AK, CO, TX, ID
Lung	72	16	22–110	WV, KY, ME, FL, AR	UT, CO, NM, AK, CA

All values are state-level, taken as the average of all years under study. Incidence is cases per 100,000 people (breast cancer per 100,000 women, prostate cancer per 100,000 men).

Malpractice payout rate and cancer risk factors also varied among states. Malpractice payout rate was highest in New York, with 9 payouts per 100,000 people, and lowest in Alabama, with one payout per 100,000 people. Further details on malpractice payout rate and cancer risk factors are presented in **Table 2**.

**Table 2:** Variation in cancer risk factors among states.

	Mean	Standard Deviation	Range	Top Decile States	Bottom Decile States
Malpractice payout rate (payouts per 100,000 people)	4	2	1–9	NY, PA, DC, LA, NJ	AL, WI, MN, ID, NC
Age (mean, years)	45	1	42–48	FL, WV, ME, PA, MT	UT, AK, TX, CA, GA
Smoking (percent adult population smokers)	21%	3%	12–29%	KY, WV, IN, OK, MO	UT, CA, HI, CT, MA
Obesity (percent population BMI>30)	24%	3%	18–30%	MS, WV, AL, LA, KY	CO, MA, HI, CT, MT
Income (median household, dollars)	47,000	7,000	35,000–61,000	MD, NH, NJ, CT, AK	MS, AR, WV, LA, MT
Poverty (percent population in poverty)	12%	3%	6–19%	MS, NM, LA, DC, AR	NH, MN, CT, NJ, MD
Insurance (percent population <65 years old with health insurance)	85%	4%	74–93%	MA, HI, MN, DE, DC	TX, LA, NM, NV, MS

All values are state-level, taken as the average of all years under study.

### Univariate analysis

**Fig 2:**
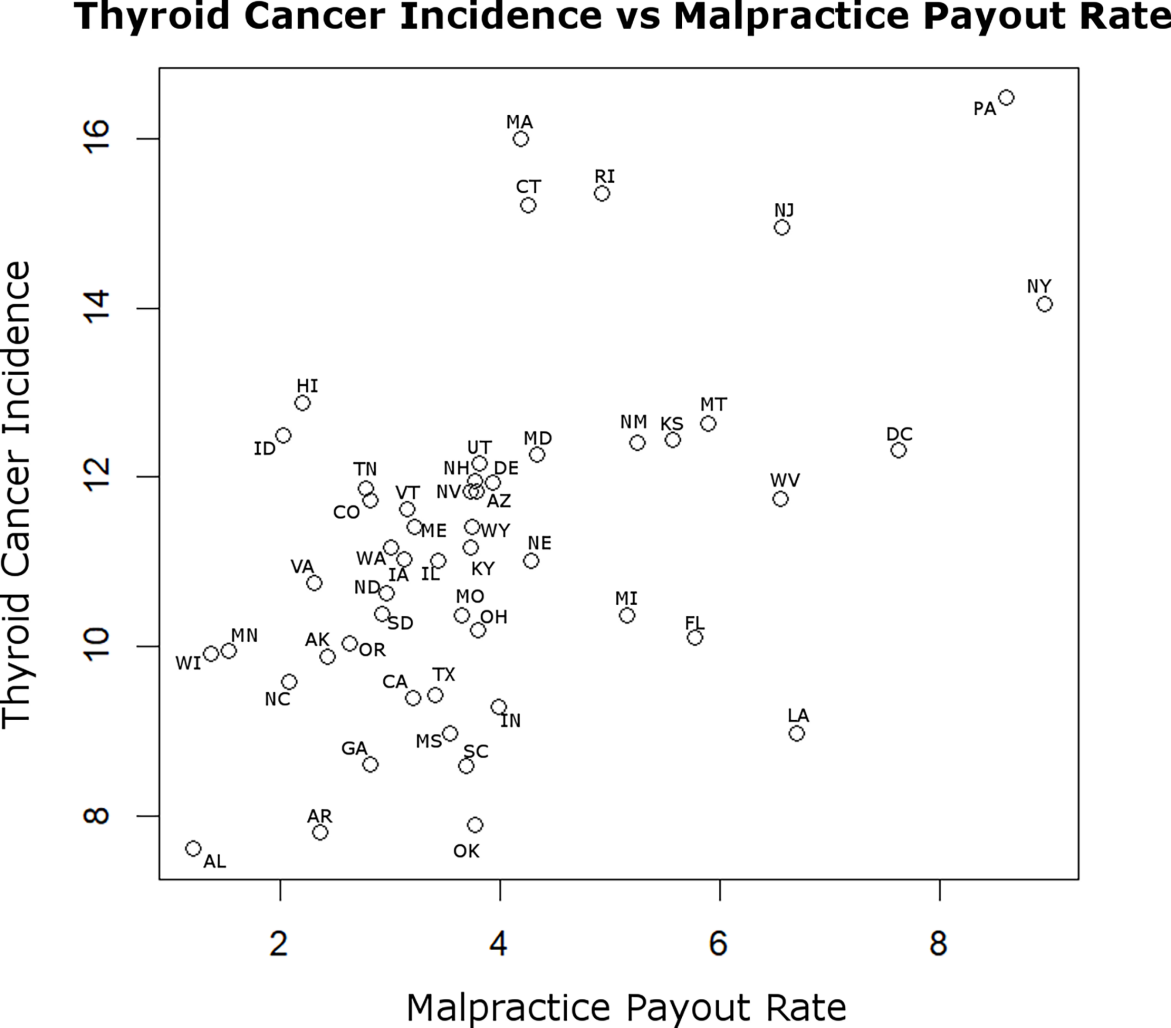
Thyroid cancer incidence versus malpractice payout rate. Values are average for each state from all years under study. States with higher malpractice payout rate had higher incidence of thyroid cancer (r = 0.51, *P* = 0.009, Spearman).

**Fig 1** demonstrates the correlations among all variables, each taken as the average in each state for all years under study (complete correlation matrix available as **S1 Table**). The figure demonstrates several themes. First, states with higher malpractice payout rate had higher incidence of thyroid cancer (r = 0.51, p = 0.009, Spearman), but malpractice payout rate associated with no other variable. A scatterplot demonstrating the association between malpractice payout rate and thyroid cancer incidence is presented in **Fig 2**, with individual states labelled. Second, markers of socioeconomic status tended to correlate with one another in the expected directions, and there were associations between cancer incidences and markers of socioeconomic status. That is, states with higher income tended to have higher rates of insurance coverage and lower rates of poverty, prompting us to create the healthcare access metric described above (Material and Methods). States with higher insurance coverage had higher rates of thyroid and breast cancer, while states with lower income had higher rates of colon cancer, all keeping with prior studies of socioeconomic status and cancer incidence[13, 24–26]. Third, smoking and obesity were highly positively correlated with each other, and negatively correlated with income, keeping with known associations[27]. Additionally, lung cancer incidence was higher in states with greater smoking and obesity, keeping with known effects of smoking on lung cancer risk[28]. Thyroid cancer incidence was higher in states with lower obesity. Last, states with older populations had higher rates of breast, prostate, colon, and lung cancer, but not thyroid cancer.

### Multivariate analysis

Results of the multivariate analysis are presented in **Table 3**. To summarize, multivariate analysis showed a significant positive association between thyroid cancer incidence and malpractice payout rate (p<0.001), when controlling for several cancer risk factors, including healthcare access. In contrast, malpractice payout rate did not significantly associate with the incidence of any other cancer type under study, though the association with colon cancer incidence approached statistical significance (p = 0.07). The associations between malpractice payout rate and the remaining three cancer types were all highly non-significant (p = 1).

**Table 3:** Multivariate models for all cancer types.

	Coefficient Estimate	SE Coefficient Estimate	*P* value	
**Thyroid**				
Year	3.2	0.11	**<0.001**	
Malpractice payout rate	8.6	1.5	**<0.001**	
Age	1.5	1	0.75	
Smoking	-0.7	0.36	0.26	
Healthcare access	5.5	1.4	**<0.001**	
**Breast**				
Year	-0.1	0.15	1	
Malpractice payout rate	2.8	2.3	1	
Age	15	1.6	**<0.001**	
Smoking	0.05	0.56	1	
Healthcare access	9.9	2.1	**<0.001**	
**Prostate**				
Year	-1.9	0.33	**<0.001**	
Malpractice payout rate	5.9	4.6	1	
Age	17	3.1	**<0.001**	
Smoking	-1.5	1.1	0.8	
Healthcare access	3.3	4.2	1	
**Colon**				
Year	-1.9	0.12	**<0.001**	
Malpractice payout rate	5.6	2.3	0.07	
Age	13	1.6	**<0.001**	
Smoking	1.8	0.56	**0.006**	
Healthcare access	3.6	2.1	0.44	
**Lung**			
Year	-0.0022	0.11	1
Malpractice payout rate	3	3.3	1
Age	20	2.2	**<0.001**
Smoking	5.5	0.8	**<0.001**
Healthcare access	2.1	3	1

Five models were created, one for each cancer type, using the same set of covariates. Cancer incidence was the dependent variable in all models. Models were designed, and presented here, to show the association between cancer incidences and covariates *among different states*. Thus, coefficient estimates presented here are the “between” version for the given variable produced by the multilevel models. The full models, including “within” coefficients are present in S1 Table. In each model, cancer incidence was transformed as arcsin($\sqrt{x}$), so coefficient estimates are not easily converted to hazard ratios. However, coefficients can be directly compared between cancer types, to give relative size differences. Coefficient estimates are 10^−4^. SE = standard error.

The multivariate models showed many other significant associations, as seen on univariate analysis and keeping with prior studies. Thyroid cancer incidence was higher in states with greater healthcare access (p<0.001). Breast cancer incidence was higher in states with older population (p<0.001) and greater healthcare access (p<0.001). Prostate cancer incidence was higher in states with older populations (<0.001). Colon cancer was more common in states with older populations (p<0.001) and higher rates of smoking (p = 0.006). Lung cancer was also more common in states with older populations (p<0.001) and higher rates of smoking (p<0.001). Thyroid cancer was more common in later calendar years, while prostate and colon cancer were less common in later calendar years (all p<0.001), keeping with national incidence data[1]. Complete model output is presented in **S2 Table**.

## Discussion

In this paper, we have shown that states with higher malpractice risk have higher incidence of thyroid cancer, when controlling for healthcare access and other risk factors. In contrast, malpractice payout rate did not correlate with incidence of any other cancer type under study, including cancers of the breast, prostate, colon, and lung. The findings suggest malpractice climate may be a social determinant of being diagnosed with thyroid cancer. Indeed, there are several established social determinants of cancer risk, which have complex behavioral and biological associations. For example, people with lower socioeconomic status have higher incidence of colon cancer, largely because they are more likely to be obese, smoke, and have physically inactive lifestyles, known risk factors for colon cancer[29–31]. Similarly, lung cancer is more common in those with lower socioeconomic status, much of this attributable to higher rates of smoking[32]. In contrast, men with higher socioeconomic status are more likely to be diagnosed with prostate cancer[33], in large part because they have greater healthcare access, which leads to more diagnostic testing (i.e. PSA screening and prostate biopsy) and thus more frequent cancer overdiagnosis[34]. Healthcare access does not account for this entire increase in prostate cancer incidence[34], however, suggesting other mechanisms may be operative. Breast cancer is similar. Women with higher socioeconomic status are more likely to be diagnosed with breast cancer, partly because they have greater healthcare access (including screening mammography) and thus greater risk of overdiagnosis, and partly because they tend to have their first child later in life and fewer children overall, known biological risk factors for breast cancer[35]. Social determinants of health are thus important in understanding cancer incidence. If malpractice climate is a social determinant of thyroid cancer risk, it may partially explain the increase in thyroid cancer incidence seen in the past several decades.

We suspect the association between thyroid cancer incidence and malpractice climate relates to the types of lawsuits pursued against physicians, and the tactics physicians employ to defend against them. Specifically, a major source of malpractice litigation against physicians relating to cancer is delayed diagnosis[36]. In these cases, the plaintiffs’ attorneys typically pursue the legal theory of negligence. That is, the attorney asserts the defendant physician acted negligently by failing to follow the standard of care[37], care a competent physician would have provided to a patient presenting similarly to the plaintiff[38]. The standard of care may be clinical management or pathologic diagnosis. In keeping with this, in the cancer context, primary care physicians are typically sued for failing to order appropriate tests, such as serum PSA or thyroid ultrasound with FNA, or failing to refer to appropriate specialists, in patients later diagnosed with advanced disease[39–41]. Pathologists are typically sued for failing to diagnose cancer in sampled material, either by failing to see it on the slides, or by misclassifying a tumor as benign, when the patient is later found to have advanced disease[42]. In the context of thyroid tumors, we believe physicians defend against these risks with two main strategies: increased use of thyroid ultrasound and FNA by clinical physicians, and lowered diagnostic thresholds for thyroid cancer among pathologists.

Regarding the first proposed defensive strategy–increased use of thyroid ultrasound and FNA—we note that delayed diagnosis accounts for the majority of malpractice lawsuits relating to thyroid cancer[39]. The paper describing this finding explicitly recommends liberal use of thyroid ultrasound with FNA to reduce tort risk[39]. Indeed, thyroid ultrasound and FNA are being used increasingly, with use rising 5-fold and 7-fold, respectively, between the years 2000 and 2012[5]. Physicians who practice in states with higher tort risk tend to defend more aggressively against it, often by ordering a greater number of diagnostic tests[14]. This practice likely extends to testing thyroid nodule with ultrasound and FNA, with a resultant increase in thyroid cancer overdiagnoses.

The second proposed strategy–lowered diagnosed thresholds among pathologists–is more subtle. Diagnostic dilemmas challenge pathologists evaluating all organ systems, but these challenges have unique clinical and epidemiological consequences in the thyroid. That is, while diagnosis of the most common type of thyroid cancer (classic papillary thyroid carcinoma) is well-defined and reproducible among pathologists, a non-trivial number of tumors fall into a diagnostic grey zone, where the distinction between carcinoma and a benign tumor is subjective and inadequately reproducible among pathologists[43, 44]. Evidence indicates that malpractice fears drive pathologists to lower their diagnostic thresholds, specifically in these equivocal cases. For example, survey based studies have shown that fear of litigation influences pathologists to favor malignant diagnoses in equivocal breast and melanocytic lesions[15, 16]. It is thus possible that pathologists who practice in states with high tort risk disproportionately favor malignant diagnoses in borderline thyroid cases, resulting in a larger number of reported cancers. These borderline cases are common, creating great latitude to respond to malpractice risk.

It is interesting that malpractice payout rate was not associated with the incidence of breast, prostate, colon, or lung cancer. We suspect this relates to cancer screening programs, the natural history of these cancer types, and histomorphologies. Screening for prostate cancer, breast cancer, and colon cancer was common in the years under study[19]. Thus, physicians required no further incentive to work up patients for these cancer types: standard of care dictated most patients of a specific age should be evaluated, shielding the physician from lawsuits. Lung cancer is extremely aggressive, with five year survival less than 20%[1]. Thus, asymptomatic patients would not stay asymptomatic for long, narrowing the window to miss a cancer diagnosis. Regarding histomorphology, compared to the thyroid, there are fewer situations in the prostate, breast, colon, and lung in which a pathologist must subjectively distinguish a benign tumor from a malignant one. Pathologists thus have little incentive, and little leeway, to lower thresholds in these other organs. Colon cancer is perhaps an exception, because it is difficult and subjective to distinguish intramucosal carcinoma (considered in situ) from early stage invasive colon cancer[45]. It is possible tort risk influences this decision, though of insufficient magnitude to be discovered in our study. A subset of breast lesions may have equivocal histologic features as well. However, these tend to be non-invasive lesions, such as atypical ductal hyperplasia vs ductal carcinoma in situ[46], neither of which would go to the cancer registries as invasive carcinoma.

In addition to defensive medicine, it is possible that malpractice payout rate acted as a proxy for another, unmeasured variable that affects thyroid cancer incidence. We went to great lengths to include described risk factors for thyroid cancer, arguing malpractice risk is not a proxy for one of these. However, it is entirely possible that malpractice risk is a proxy for an unknown biological risk factor. It is also possible that malpractice payout rate is a proxy for another social determinant of health. For example, cultures differ among states, and it is possible that members of some cultures, most notably those in the South, are less likely to sue and less likely to seek medical treatment for non-emergent issues. Fewer lawsuits and fewer thyroid cancer diagnoses could result. In contrast, cultures from the North Atlantic may be more likely to sue and more likely to seek medical treatment.

In conclusion, we have shown that malpractice climate may be a social determinant of thyroid cancer risk. If further study shows that high-risk malpractice climates indeed increases risk of thyroid cancer overdiagnosis, policy measures may be required to mitigate this risk. In this vein, measures to reduce tort risk may not be mere cost-saving measures. They may serve to reduce the risk of thyroid cancer overdiagnosis.

## Supporting information

S1 Table Complete correlation matrix used to generate Fig 1. (XLSX)

S2 Table Complete output of multivariate models, including between and within versions. (DOCX)
