# Supplementary material for: Evidence of a positive association between malpractice climate and thyroid cancer incidence in the United States
Source: PLoS One. 2018 Jul 18;13(7):e0199862. doi: 10.1371/journal.pone.0199862 (PMC6051569; doi:10.1371/journal.pone.0199862)
Supplement: S2 Table — (DOCX) [file pone.0199862.s002.docx]

Table S2a. Thyroid cancer – complete model

|  | **Coefficient estimate** | **Standard Error** | **Corrected p value** |
| --- | --- | --- | --- |
| **Intercept** | 0.01813 | 0.005350 | 0.007 |
| **Year** | 0.000318 | 0.000011 | <.001 |
| **Malpractice rate between** | 0.000855 | 0.000150 | <.001 |
| **Malpractice rate within** | 0.000070 | 0.000076 | 1 |
| **Age between** | 0.000146 | 0.000102 | 0.75 |
| **Age within** | -0.00003 | 0.000054 |  |
| **Smoking between** | -0.00007 | 0.000036 | 0.26 |
| **Smoking within** | -0.00006 | 0.000015 | <.001 |
| **Healthcare access between** | 0.000548 | 0.000137 | <.001 |
| **Healthcare access within** | 0.000265 | 0.000087 | 0.013 |

Table S2b. Breast cancer – complete model

|  | **Coefficient estimate** | **Standard Error** | **Corrected p value** |
| --- | --- | --- | --- |
| **Intercept** | -0.02647 | 0.008250 | 0.012 |
| **Year** | -0.00001 | 0.000015 | 1 |
| **Malpractice rate between** | 0.000277 | 0.000232 | 1 |
| **Malpractice rate within** | 3.388E-7 | 0.000110 | 1 |
| **Age between** | 0.001498 | 0.000157 | <.001 |
| **Age within** | 0.000636 | 0.000078 | <.001 |
| **Smoking between** | 5.469E-6 | 0.000056 | 1 |
| **Smoking within** | 0.000059 | 0.000022 | 0.046 |
| **Healthcare access between** | 0.000992 | 0.000211 | <.001 |
| **Healthcare access within** | -0.00017 | 0.000127 | 0.9 |

Table S2c – Prostate cancer, complete model

|  | **Coefficient estimate** | **Standard Error** | **Corrected p value** |
| --- | --- | --- | --- |
| **Intercept** | -0.02703 | 0.01625 | 0.5 |
| **Year** | -0.00019 | 0.000033 | <.001 |
| **Malpractice rate between** | 0.000585 | 0.000456 | 1 |
| **Malpractice rate within** | 0.000316 | 0.000235 | 0.9 |
| **Age between** | 0.001716 | 0.000309 | <.001 |
| **Age within** | -0.00003 | 0.000166 | 1 |
| **Smoking between** | -0.00015 | 0.000111 | 0.8 |
| **Smoking within** | -0.00018 | 0.000048 | 0.001 |
| **Healthcare access between** | 0.000333 | 0.000415 | 1 |
| **Healthcare access within** | 0.000783 | 0.000271 | 0.02 |

Table 2d – Colon cancer, complete model

|  | **Coefficient estimate** | **Standard Error** | **Corrected p value** |
| --- | --- | --- | --- |
| **Intercept** | -0.03141 | 0.008157 | 0.002 |
| **Year** | -0.00019 | 0.000012 | <.001 |
| **Malpractice rate between** | 0.000564 | 0.000229 | 0.07 |
| **Malpractice rate within** | 0.000141 | 0.000082 | 0.44 |
| **Age between** | 0.001291 | 0.000155 | <.001 |
| **Age within** | -8.67E-6 | 0.000058 | 1 |
| **Smoking between** | 0.000180 | 0.000056 | 0.006 |
| **Smoking within** | 8.662E-6 | 0.000017 | 1 |
| **Healthcare access between** | 0.000357 | 0.000209 | 0.44 |
| **Healthcare access within** | -0.00014 | 0.000095 | 0.75 |

Table S2e – Lung cancer, complete model

|  | **Coefficient estimate** | **Standard Error** | **Corrected p value** |
| --- | --- | --- | --- |
| **Intercept** | -0.06876 | 0.01173 | <.001 |
| **Year** | -2.23E-7 | 0.000011 | 1 |
| **Malpractice rate between** | 0.000301 | 0.000330 | 1 |
| **Malpractice rate within** | 0.000204 | 0.000078 | 0.045 |
| **Age between** | 0.001953 | 0.000223 | <.001 |
| **Age within** | 0.000168 | 0.000055 | 0.012 |
| **Smoking between** | 0.000546 | 0.000080 | <.001 |
| **Smoking within** | -0.00003 | 0.000016 | 0.16 |
| **Healthcare access between** | 0.000207 | 0.000300 | 1 |
| **Healthcare access within** | 0.000016 | 0.000090 | 1 |
